# Depression diagnosis based on electroencephalography power ratios

**DOI:** 10.1002/brb3.3173

**Published:** 2023-07-21

**Authors:** Jinwon Chang, Yuha Choi

**Affiliations:** ^1^ Korean Minjok Leadership Academy Hoengseong‐gun Gangwon‐do Republic of Korea

**Keywords:** alpha/beta ratio, depression, electroencephalography, neural network

## Abstract

**Background:**

Depression is a common mental disorder that impacts millions of people across the world. However, its diagnosis is difficult due to the dependence on subjective testing. Although quantitative electroencephalography (EEG) has been investigated as a promising diagnostic tool for depression, the associated results have proven contradictory. The current study determines whether the alpha/beta (ABR), alpha/theta (ATR), and theta/beta (TBR) ratios can serve as biological markers of depression.

**Methods:**

We used open‐access EEG data from OpenNeuro to investigate power ratios in the resting state of 46 patients with depression and 75 healthy controls. Spectral data were extracted by fast Fourier transform at the theta band (4–8 Hz), alpha band (8–13 Hz), and beta band (13–32 Hz). Neural network, logistic regression, and receiver operating characteristic (ROC) curves were used to assess the diagnostic accuracies of each suggested index. Additionally, the cutoff point, sensitivity, specificity, positive predictive value, and negative predictive value at the maximized Youden index were compared for each variable.

**Results:**

Decreased anterior frontal, frontal, central, parietal, occipital, and temporal ABR and decreased central and parietal TBR were observed in the depression group. The area under the curve of the ROC curves further revealed that these ratios could all effectively differentiate depression. In particular, the central, frontal, and parietal ABR exhibited high discrimination scores. Multiple logistic regression analysis demonstrated that the Beck Depression Inventory and Spielberger Trait Anxiety Inventory scores, as well as the probability of depression, increased with a decrease in the central ABR. Moreover, neural network analysis revealed that the global ABR was the most effective index for diagnosing depression among the three global EEG power ratios.

**Conclusions:**

The central, frontal, and parietal ABR represent potential biomarkers to differentiate patients with depression from healthy controls.

## Graphical abstract

This study investigated the potential of EEG power ratios as biological markers for diagnosing depression. The results suggest that decreased central, frontal, and parietal alpha/beta ratios could effectively differentiate patients with depression from healthy controls. These findings may contribute to the development of more effective and efficient methods for diagnosing depression.

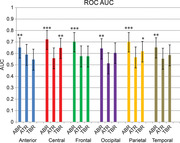

## 1 INTRODUCTION

Depression is a common mental disorder that can severely affect mood, behavior, and personality. According to the World Health Organization (WHO) (2017), the number of patients with depression has exceeded 300 million globally. Moreover, the recent COVID‐19 pandemic has increased the prevalence of anxiety, depression, and stress across the world (Lakhan et al., 2020). However, the clinical diagnosis of depression is highly dependent on subjective cognitive tests and physician judgment. Meanwhile, diagnosing depression using a dimensional approach has proven even more challenging. Therefore, a more effective diagnostic method using objective biological markers is needed to facilitate the early treatment of patients.

Magnetic resonance imaging (MRI) and electroencephalography (EEG) have been proposed as alternatives to current cognitive tests. More specifically, several imaging technologies, including functional MRI (resonance), high‐resolution structural imaging (3D‐T1), and diffusion tensor imaging, have been evaluated as potentially effective means to detect neurobiological markers of major depressive disorder (MDD) (Helm et al., 2018). However, these methods are expensive and restricted to early diagnosis.

EEG is an inexpensive, noninvasive, and relatively convenient diagnostic tool for assessing the cognitive state of individuals. However, most patterns of EEG abnormalities in patients with depression are inconsistent (de Aguiar Neto & Rosa, 2019). Among the spectral‐, signal‐, and network‐based features, including band power in each frequency range, alpha asymmetry, randomness, and functional connectivity, only results related to the theta and Higuchi's fractal dimensions have proven relatively consistent in the context of depression (de Aguiar Neto & Rosa, 2019; Pizzagalli et al., 2006). However, analyses using relative and absolute theta power have generated differing results. That is, although a significant difference has been reported in absolute theta power between patients with depression and healthy controls (CTLs), no significant differences have been detected using relative theta power (Newson & Thiagarajan, 2019). In addition, one study criticized Higuchi's fractal dimension method as being mathematically incorrect (Martišek, 2022). Hence, new neurobiological markers, such as the spectral power ratio between the slow and fast bands, are needed for the effective diagnosis of depression. The ratio approach to evaluate spectral power provides the effective brain index with easier calculations compared to nonlinear EEG features. Furthermore, confusion and inconsistency between absolute and relative power value like the example of theta band could be eradicated when the ratio between bands is used. By connecting two possibly affected wave bands together, the ratio approach can also amplify the subtle abnormalities in the depressed brain so that they could easily be detected.

The frequency band power ratios reflect the activity and state of the brain, with the slow and fast band ratios used to assess various cognitive states. Alpha oscillation manifests neuronal inactivity, relaxation, and goal‐related emotion, whereas beta oscillation is associated with expectancy, anxiety, and internal control, and theta oscillation reflects emotional processing (Abhang et al., 2016; Aftanas & Golocheikine, 2001; Aftanas et al., 2002; Coan & Allen, 2004; Freeman & Quian Quiroga, 2012; Rao, 2013). Combination of such different frequency ranges enables an acute assessment of complex cognitive states. For instance, the alpha/beta (ABR) and theta/beta (TBR) ratios have been successfully applied to determine the degree of stress (Yi Wen & Mohd Aris, 2020). Meanwhile, the ATR or theta/alpha ratios can successfully differentiate patients with Alzheimer's, Parkinson's, and other Lewy body diseases from CTLs (Baik et al., 2022; Jaramillo‐Jimenez et al., 2021; Özbek et al., 2021; Schmidt et al., 2013; Zawiślak‐Fornagiel et al., 2023). Additionally, the TBR is useful for distinguishing Lewy body diseases from Alzheimer's disease (Baik et al., 2022). Accordingly, spectral power ratios may also be capable of effectively differentiating between patients with depression and healthy individuals, given that several cognitive abnormalities are associated with depression. ATR might be able to detect emotional fluctuation and impaired cognitive ability, which is highly correlated with depressive states (Beaujean et al., 2013; Hammen, 2005). In addition, ABR and TBR might be able to capture emotional stress and anxiety commonly found in depression group (Hammen, 2005; Yi Wen & Mohd Aris, 2020). We, therefore, hypothesize that, by using a machine learning approach, the ATR, TBR, and ABR can effectively differentiate patients with depression from CTLs.

## 2 METHODS

### 2.1 Participants

This study used open‐access datasets from OpenNeuro (Cavanagh, 2021) comprising 122 participants whose cognitive state was assessed using the Beck Depression Inventory (BDI) and the Spielberger Trait Anxiety Inventory (STAI) in mass and laboratory assessments. According to the original study (Cavanagh et al., 2019), participants aged 18–25 years with no history of head trauma or seizures and no current psychoactive medication use were included in the study. If the BDI scores were consistently high, a Structured Clinical Interview for Depression was conducted to determine whether the participant had MDD (Cavanagh et al., 2019). The MDD group (*n* = 21) was not separated but incorporated in the depression group in the current study as it used a dimensional approach for depression. Among the 122 participants, one was excluded due to unstable BDI scores between mass and laboratory assessments. Participants with BDI scores >13 were considered depressed (*n* = 46; 34 females). CTLs had stable low BDI scores (<7) and no self‐reported history or symptoms of anxiety disorder (*n* = 75, 40 females). According to the original study (Cavanagh et al., 2019), all participants provided written informed consent as approved by the University of Arizona. The current study was approved by the public institutional review board designated by the Ministry of Health and Welfare (P01‐202304‐01‐003).

### 2.2 EEG acquisition

Scalp EEG was measured with 64 Ag/AgCl electrodes in a 10/10 system using a Synamps2 system (band‐pass filter: 0.5–100 Hz, sampling rate: 500 Hz, impedances <10 kΩ; the online reference was a single channel placed between Cz and CPz) (Cavanagh et al., 2019). All raw EEG data were recorded for 6 min per session in the resting state, and two recording sessions per participant were conducted: one before the task described in the original study (Cavanagh et al., 2019) and the other after the task. Each EEG session included eyes‐closed and eyes‐open states and lasted 1 min. Only the resting‐state EEG obtained before the task was used to avoid the probable interference effect of the task on the resting‐state EEG.

### 2.3 EEG preprocessing

EEGLAB was used to preprocess all EEG datasets. The EEG signals were filtered at 1 Hz, which is appropriate for independent component analysis (ICA) (Klug & Gramann, 2020). The sampling rate was downsampled to 256 Hz. The raw data were cleaned with clean raw data and ASR plugins in EEGLAB to remove bad data periods (Delorme & Makeig, 2004; Miyakoshi & Kothe, 2019). ICA was conducted, and artifacts from the eyes, muscles, line noise, and channel noise were removed. All remaining data were average‐referenced.

### 2.4 Spectral data acquisition

**FIGURE 1:**
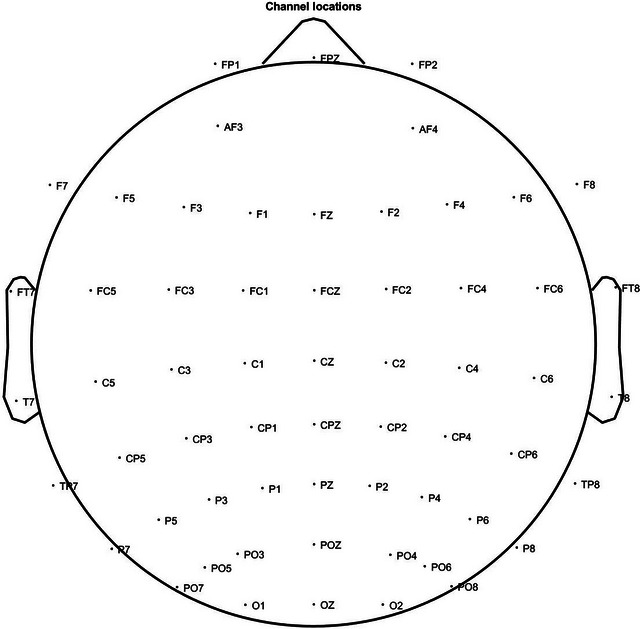
Electroencephalography (EEG) electrode placement. The electrode location is allocated on 10–20 system.

The spectral power was obtained by a fast Fourier transform (FFT) with 2048 samples for the FFT length. The absolute spectral power was extracted from each frequency band of theta (4–8 Hz), alpha (8–13 Hz), and beta (13–32 Hz). Electrodes were separated and averaged into regional groups: anterior frontal (AF3, AF4), frontal (F1, F2, F3, F4, F5, F6, F7, F8, FC1, FC2, FC3, FC4, FC5, FC6, FCz, Fp1, Fp2, Fpz, FT7, FT8, Fz), central (C1, C2, C3, C4, C5, C6, CP1, CP2, CP3, CP4, CP5, CP6, CPz, Cz), occipital (O1, O2, Oz), parietal (P1, P2, P3, P4, P5, P6, P7, P8, po3, po4, po5, po6, po7, po8, poz, Pz), and temporal (T7, T8, TP7, TP8). Electrode location is shown in Figure 1. The spectral band ratios were obtained by calculating the ABR, ATR, and TBR.

### 2.5 Neural network analysis

A neural network model was developed using the Keras package in Python. The available data comprising 120 EEG datasets each of which was extracted from each participant were partitioned into three groups: training (85 datasets), evaluation (11 datasets), and testing (24 datasets) sets, using a random sampling technique. The number of datasets for each group was determined arbitrarily, but the number of datasets for training was set to be larger than the half and three times more than that for testing. The neural network architecture consisted of an input layer comprising 60 neurons and two hidden layers comprising 40 and 20 neurons, respectively. The structure was determined empirically using the criterion of maximizing accuracy in predicting the results. Backpropagation was employed as the training method, with the network weights redetermined for every three input data points (batch size = 3). The neural network was trained for 2500 epochs to minimize errors. The sigmoid and Rectified Linear Unit activation functions were used, and the network was evaluated. An output threshold of 0.5 was set, with values of 0 and 1 used to represent normal and depressed, respectively. The performance of the network on the test data was evaluated by comparing the output results, which were transformed using the activation functions and threshold values, with the true values.

### 2.6 Statistical analyses

The statistical packages for the Social Sciences version 25.0 (IBM Corp.) and MedCalc Statistical Software version 20.218 (MedCalc Software Ltd.; https://www.medcalc.org; 2023) were used for all statistical analyses. Chi‐square and Mann–Whitney *U*‐tests were used to compare the clinical and demographic characteristics and EEG power ratios between patients with depression and CTLs. Multiple linear regression analyses with forward selection were used to determine the relationship between each lobar power ratio and the neuropsychological test scores. Age and sex were included as the covariates and factors, respectively. Multiple logistic regression using forward selection with a likelihood ratio was used to determine the association between each lobar power ratio and depression. Age and sex were included as the covariates and factors, respectively. For both regression models, an *F* probability <.05 was used as the entry, and >.10 was set as the removal level. For each lobar power ratio, receiver operating characteristic (ROC) curves and the areas under the curve (AUC) were drawn and compared using Delong's method, and binomial exact 95% AUC confidence intervals (CIs) were calculated (Delong et al., 1988). Cutoff point, sensitivity, specificity, positive predictive value (PPV), and negative predictive value (NPV) were measured at maximum Youden index after the standardization of disease prevalence to 50% (Heston, 2011; Youden, 1950). Overall, 95% CI for cutoff point and Youden index was estimated with bootstrapping method (5000 iterations, 978 seeds). Overall, 95% binomial exact CI for sensitivity, specificity, PPV, and NPV was estimated by Delong's method (Delong et al., 1988). The machine learning results using alpha/beta, alpha/theta, and theta/beta were assessed for accuracy, sensitivity, specificity, PPV, NPV, and AUC. PPV and NPV were measured after standardizing the disease prevalence to 50%. The AUC was calculated using the single‐point method (Zhang & Mueller, 2005). For the neural network, eight iterations were used for statistical analysis.

## 3 RESULTS

### 3.1 Clinical and demographic characteristics

The clinical and demographic characteristics are presented in Table 1. No significant differences were observed in age between the depression and CTL groups (*p* = .211). However, the BDI and STAI scores were higher in the depression group than in the CTL group (*p* < .001), and the depression group comprised more females than the CTL group (*p* = .02).

**TABLE 1:** Clinical and demographic characteristics of depressed patients and healthy controls.

Characteristic	Depressed	Control	*p* Value
STAI	55.76 (7.08)	31.05 (5.49)	<.001
BDI	22.22 (4.90)	1.73 (1.66)	<.001
Age	18.74 (1.19)	18.99 (1.21)	.211
Sex, female	34 (0.74)	40 (0.53)	.02
Sample size	46	75	N/A

*Note*: Data are expressed as mean (SD) or number (proportion). Chi‐square or Mann–Whitney *U*‐tests were employed as appropriate.

Abbreviations: BDI, Beck Depression Inventory; STAI, Spielberger Trait Anxiety Inventory.

### 3.2 Significant differences in the power ratios between the depression and control groups

The statistical analysis of differences in the power ratios between the depression and control groups is presented in Table 2. The Mann–Whitney *U*‐test was conducted to determine differences in lobar ABR, ATR, and TBR between the depression and control groups. The anterior frontal (*p* = .005), frontal (*p* < .001), central (*p* < .001), parietal (*p* < .001), occipital (*p* = .008), and temporal (*p* = .005) regions exhibited lower ABRs in the depression group than the CTL group. Similarly, the central (*p* = .006) and parietal (*p* = .03) TBRs were lower in the depression group than the CTL group. Meanwhile, no regions exhibited significant differences in the ATR (*p* > .05).

**TABLE 2:** Comparison of lobar power ratio between depressed patients and healthy controls.

		Depressed	Control	*p* Value
Anterior	ABR	0.95 (0.27)	1.11 (0.28)	.005
	ATR	0.75 (0.19)	0.81 (0.19)	.101
	TBR	1.43 (0.72)	1.50 (0.81)	.399
Frontal	ABR	0.96 (0.24)	1.10 (0.21)	<.001
	ATR	0.80 (0.16)	0.85 (0.16)	.165
	TBR	1.35 (0.60)	1.39 (0.39)	.172
Central	ABR	0.89 (0.21)	1.07 (0.22)	<.001
	ATR	0.95 (0.18)	0.99 (0.19)	.286
	TBR	1.00 (0.33)	1.13 (0.32)	.006
Parietal	ABR	0.98 (0.26)	1.16 (0.24)	<.001
	ATR	1.03 (0.21)	1.07 (0.18)	.221
	TBR	1.02 (0.40)	1.13 (0.31)	.03
Occipital	ABR	0.97 (0.32)	1.12 (0.28)	.008
	ATR	1.09 (0.25)	1.09 (0.21)	.81
	TBR	0.98 (0.61)	1.06 (0.33)	.055
Temporal	ABR	0.91 (0.24)	1.04 (0.27)	.005
	ATR	0.88 (0.18)	0.91 (0.16)	.315
	TBR	1.15 (0.58)	1.22 (0.63)	.116

*Note*: Data are expressed as mean (SD); Mann–Whitney *U*‐test.

Abbreviations: ABR, alpha/beta ratio; ATR, alpha/theta ratio; TBR, theta/beta ratio.

### 3.3 Effect of power ratio on neuropsychological test scores and probability of depression

The effect of the power ratio on neuropsychological test scores and the probability of depression is presented in Table 3. Multiple linear regression was conducted to determine the effect of the lobar power ratios on the BDI and STAI scores. As a result of forward selection, only the central ABR had a significant effect on the BDI scores (*p* < .001). That is, an increase in the central ABR caused a decrease in the BDI (standard beta = −.315). In addition, as a result of forward selection, only the central ABR had a significant effect on the STAI scores (*p* = .002), with an increase in the central ABR associated with a decrease in the STAI (standard beta = −.285). Sex and age did not influence either model.

**TABLE 3:** Association of lobar power ratio with neuropsychological test scores and the presence of depression.

BDI			
Central ABR	Beta (SE)	Std. beta	*p* Value
	−14.063 (3.895)	−.315	<.001
**STAI**			
Central ABR	Beta (SE)	Std. beta	*p* Value
	−16.27 (5.041)	−.285	.002
**Depression**			
Central ABR	Odds ratio		*p* Value
	.021 (.003–.158)		<.001

*Note*: Multiple logistic or linear regression was performed with forward selection (enter variables if probability <0.05 and remove if >0.1).

Abbreviations: ABR, alpha/beta ratio; BDI, Beck Depression Inventory; STAI, Spielberger Trait Anxiety Inventory.

A multiple logistic regression analysis with forward selection using the likelihood ratio was conducted to determine the effects of lobar ABR, ATR, and TBR on the probability of depression. Only the central ABR was a significant independent variable in the model (*p* < .001); an increase in central ABR was associated with a decrease in the likelihood of depression (odds ratio: .021, 95% CI: .003–.158).

### 3.4 AUC of ROC analysis for lobar power ratios and depression

**FIGURE 2:**
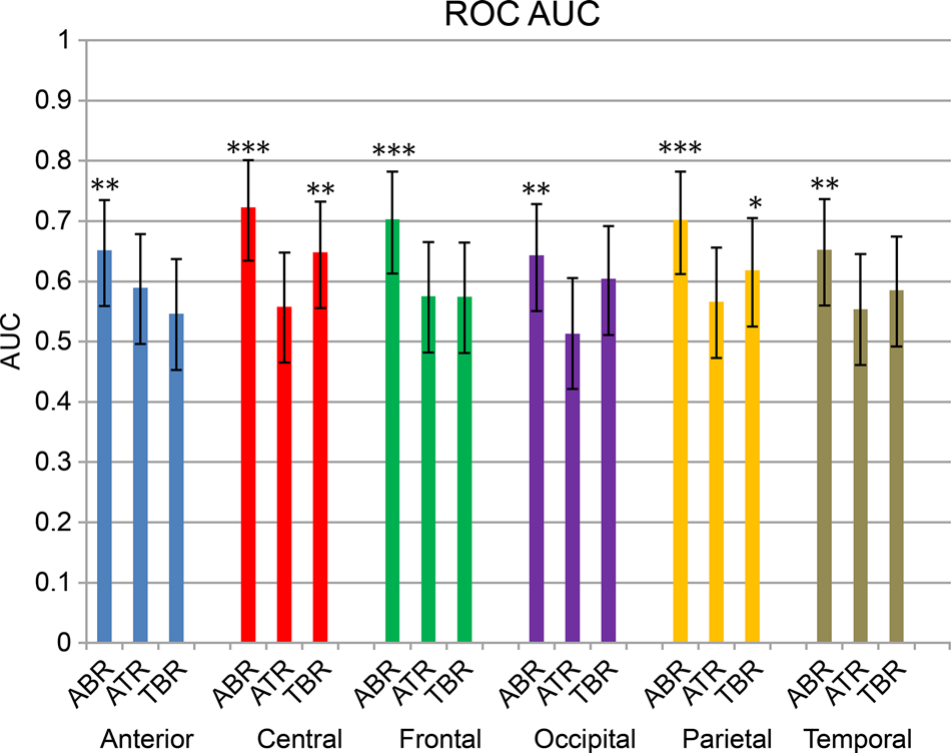
Area under the curve (AUC) of the receiver operating characteristic (ROC) curve for each lobar power ratio. Null hypothesis was set as 0.5 AUC; Delong's method was used to statistically assess whether AUC of each lobar power ratio is bigger than 0.5 AUC. Error bars represent the 95% confidence interval. *p < .05, **p < .01, ***p < .001. ABR, alpha/beta ratio; ATR, alpha/theta ratio; TBR, theta/beta ratio.

The AUC of the ROC curve for each lobar power ratio for depression is shown in Figure 2. A mean AUC > 0.7 was observed for central ABR (0.723, 95% CI: .645–.801), frontal ABR (0.703, 95% CI: .624–.782), and parietal ABR (0.702, 95% CI: .622–.782). A mean AUC = 0.6–0.7 was observed for the anterior frontal ABR (0.651 95% CI: .567–.735), central TBR (0.648 95% CI: .564–.732), occipital ABR (0.643 95% CI: .558–.728) and TBR (0.604 95% CI: .516–.692), parietal TBR (0.618 95% CI: .531–.705), and temporal ABR (0.652 95% CI: .568–.736). A mean AUC < 0.6 was observed for the anterior frontal ATR and TBR, central ATR, frontal ATR and TBR, occipital ATR, parietal ATR, and temporal ATR and TBR. Lobar power ratios with an AUC > 0.5 were anterior frontal ABR (*p* = .0034), central ABR (*p* < .0001), central TBR (*p* = .0051), frontal ABR (*p* = .0001), occipital ABR (*p* = .0069), parietal ABR (*p* = .0001), parietal TBR (*p* = .0358), and temporal ABR (*p* = .004). No significant differences were observed between the central ABR and TBR (*p* = .0912), although the central ABR had a larger AUC than the central TBR. The parietal ABR was significantly higher than the parietal TBR (*p* = .0301).

### 3.5 Representative diagnostic values of the lobar power ratios for depression

The representative diagnostic values of the lobar power ratio for depression are shown in Table 4. Optimal cutoff points for sensitivity, specificity, PPV, and NPV were suggested using the Youden index method. The NPV and PPV were standardized at a disease prevalence of 50%. These values can be used for comparison in future studies.

**TABLE 4:** Representative diagnostic accuracy values for lobar power ratios.

		Cutoff	Sensitivity	Specificity	Youden index	PPV	NPV
Anterior	ABR	≤1.02 (0.77–1.28)	65.2 (49.8–78.6)	61.3 (49.4–72.4)	0.266 (0.103–0.381)	62.8 (54.2–70.6)	63.8 (53.3–73.1)
	ATR	≤0.82 (0.77–1.14)	73.9 (58.9–85.7)	48.0 (36.3–59.8)	0.219 (0.098–0.351)	58.7 (51.9–65.2)	64.8 (51.7–76.0)
	TBR	≤0.98 (0.82–1.60)	28.3 (16.0–43.5)	89.3 (80.1–95.3)	0.176 (0.080–0.250	72.6 (54.3–85.5)	55.5 (50.5–60.3)
Central	ABR	≤0.87 (0.70–0.99)	50.0 (34.9–65.1)	88.0 (78.4–94.4)	0.380 (0.188–0.483)	80.6 (67.9–89.1)	63.8 (56.6–70.4)
	ATR	≤0.96 (0.84–1.22)	60.9 (45.4–74.9)	54.7 (42.7–66.2)	0.155 (0.081–0.213)	57.3 (48.9–65.4)	58.3 (48.0–67.9)
	TBR	≤0.95 (0.81–1.31	54.4 (39.0–69.1)	76.0 (64.7–85.1)	0.304 (0.148–0.444)	69.4 (58.3–78.6)	62.5 (54.2–70.0)
Frontal	ABR	≤0.99 (0.85–1.04)	65.2 (49.8–78.6)	70.7 (59.0–80.6)	0.359 (0.156–0.466)	69 (59.6–77.0)	67 (57.1–75.6)
	ATR	≤0.80 (0.65–0.93)	58.7 (43.2–73.0)	61.3 (49.4–72.4)	0.200 (0.092–0.314)	60.3 (51.1–68.8)	59.8 (50.2–68.7)
	TBR	≤0.97 (0.81–1.31	30.4 (17.7–45.8)	93.3 (85.1–97.8)	0.238 (0.148–0.444)	82 (63.8–92.2)	57.3 (52.3–62.1)
Occipital	ABR	≤1.09 (0.84–1.45)	71.7 (56.5–84.0)	54.7 (42.7–66.2)	0.264 (0.111–0.373)	61.3 (53.8–68.3)	65.9 (53.9–76.2)
	ATR	≤1.35 (0.84–1.22)	78.3 (63.6–89.1)	6.7 (2.2–14.9)	0.151 (0.082–0.213)	45.6 (41.6–49.7)	23.5 (10.1–45.7)
	TBR	≤0.75 (0.70–1.18)	39.1 (25.1–54.6)	92 8 (83.4–97.0)	0.311 (0.167–0.447)	83.67 (67.7–91.9)	60.2 (54.3–65.8)
Parietal	ABR	≤1.03 (0.88–1.19)	63.0 (47.5–76.8)	72.6 (60.4–81.8)	0.356 (0.162–0.464)	69.2 (59.5–77.5)	66.1 (56.6–74.5)
	ATR	≤1.01 (1.00–1.36)	56.5 (41.1–71.1)	70.7 (59.0–80.6)	0.272 (0.137–0.446)	65.8 (55.5–74.8)	61.9 (53.1–70.0)
	TBR	≤0.86 (0.77–1.27)	41.3 (27.0–56.8)	89.3 (80.1–95.3)	0.306 (0.159–0.449)	79.5 (64.9–89.0)	60.3 (54.1–66.3)
Temporal	ABR	≤0.85 (0.76–1.12)	47.8 (32.9–63.1)	80.0 (69.2–88.4)	0.278 (0.114–0.392)	70.5 (58.1–80.5)	60.5 (53.2–67.4)
	ATR	≤0.77 (0.72–1.03)	34.8 (21.4–50.2)	85.3 (75.3–92.4)	0.201 (0.108–0.306)	70.3 (54.7–82.3)	56.7 (50.9–62.2)
	TBR	≤1.00 (0.86–2.15	52.2 (36.9–67.1)	70.7 (59.0–80.6)	0.228–(0.1100.350)	64 (53.2–73.6)	59.6 (51.4–67.4)

*Note*: Data are expressed as mean (95% CI). Cutoff point, sensitivity, specificity, PPV, and NPV were measured at maximum Youden index after standardization of disease prevalence to 50%; 95% CI for cutoff point and Youden index was estimated with bootstrapping method (5000 iterations, 978 seeds); 95% binomial exact CI for sensitivity, specificity, PPV, and NPV was estimated by Delong's method.

Abbreviations: ABR, alpha/beta ratio; ATR, alpha/theta ratio; CI, confidence interval; NPV, negative predictive value; PPV, positive predictive value; TBR, theta/beta ratio.

### 3.6 Representative diagnostic values of the neural network for depression

The representative diagnostic values of the neural network for depression are presented in Table 5. Accuracy, sensitivity, specificity, PPV, NPV, and AUC are presented with a 95% CI. PPV and NPV were estimated after standardization of disease prevalence at 50%. The ABR showed a significantly higher mean accuracy, specificity, and PPV than the ATR. Compared with the TBR, the ABR exhibited higher diagnostic discrimination power in all aspects, including accuracy, sensitivity, specificity, PPV, NPV, and AUC. Similarly, the ATR showed a higher accuracy, sensitivity, PPV, NPV, and AUC than the TBR.

**TABLE 5:** Representative values of neural network diagnostic accuracy.

	ABR		ATR		TBR	
	Mean	95% CI	Mean	95% CI	Mean	95% CI
Accuracy	67.71^a^, ^b^	64.10–71.31	58.85^a^, ^c^	55.40–62.31	49.48^b^, ^c^	48.25–50.71
Sensitivity	56.94^b^	53.66–60.23	59.72^c^	47.62–71.82	36.36^b^, ^c^	32.30–40.43
Specificity	74.17^a^, ^b^	67.89–80.44	59.17^a^	49.54–68.79	62.5^b^	60.23–64.77
PPV	69.31^a^, ^b^	64.12–74.51	59.60^a^, ^c^	56.81–62.38	49.09^b^, ^c^	44.96–53.24
NPV	63.23^b^	61.11–65.35	60.29^c^	56.24–64.34	49.59^b^, ^c^	47.24–51.94
AUC	0.717^b^	MIN–MAX	0.633^c^	MIN–MAX	0.5^b^, ^c^	MIN‐MAX
		0.656–.778		.594–.671		.5–.5

*Note*: Accuracy, sensitivity, specificity, PPV, NPV, and AUC are presented at disease prevalence of 50%. AUCs of receiver operating characteristic curve were calculated by single‐point method.

Abbreviations: ABR, alpha/beta ratio; ATR, alpha/theta ratio; AUC, area under curve; CI, confidence interval; NPV, negative predictive value; PPV, positive predictive value; TBR, theta/beta ratio.

^a^ Significantly different in the comparison between ABR and ATR.

^b^ Significantly different in the comparison between ABR and TBR.

^c^ Significantly different in the comparison between ATR and TBR.

## 4 DISCUSSION

The current study evaluated whether ABR, ATR, and TBR could effectively discriminate patients with depression from CTLs. Before examining the spectral power ratios, clinical and demographic characteristics of the depression and control groups were assessed. Although both groups had similar age ranges, the depression group had a significantly higher number of females. As depression is more common and severe in women, this difference should be considered in future studies (World Health Organization [WHO], 2017). Significance tests revealed that all lobar ABRs decreased in the depression group compared to the CTL group, whereas no differences in the ATRs, and a smaller TBR, were observed only in the central and parietal lobes. In particular, in the regression model, a decreased central ABR caused an increase in BDI and STAI scores and the likelihood of depression. In the AUC comparison, all lobar ABRs and central and parietal TBRs exhibited significant AUC discrimination scores (>0.5), which was consistent with the results of the significance test. In particular, central, frontal, and parietal ABRs showed AUC discrimination scores >0.7, which represents a “good” indicator of depression (Simundic, 2012).

Representative diagnostic values of each lobar power ratio, including the cutoff point, sensitivity, specificity, PPV, NPV, and Youden index, are presented at optimal cutoff points determined by the maximized Youden index with a standardized disease prevalence of 50% (Heston, 2011; Youden, 1950). However, these representative values do not represent the diagnostic accuracy of real tests. In the current study, although the optimal cutoff points were determined using the Youden index, this method assumes equal sensitivity and specificity weights, ignoring the cost of decisions, and pursues only the maximized sum of sensitivity and specificity, which is clearly not the case in real‐world settings. In addition, disease prevalence was standardized at 50% to estimate the PPV and NPV; however, the actual prevalence was much lower. Nevertheless, these standardized values enable a simple and effective comparison with other biomarkers of depression, unlike the non‐standardized values, for which the actual prevalence or pretest probability is not fixed. As such, it is difficult to compare non‐standardized values under the same conditions. In addition, using neural networks, the current study presents the representative diagnostic values of combined ABR, ATR, and TBR. ABR was more effective in detecting depression than ATR and TBR. The ATR was also more effective than TBR for diagnosing depression, whereas the ATR was inferior to the ABR in terms of accuracy, specificity, and PPV.

Although it is difficult to determine the precise role of the ABR, ATR, and TBR in depression due to the high state‐dependent characteristics of EEG power, previous studies have provided empirical evidence to predict their functions. For instance, the decreased ABRs and TBRs in the depression group may represent increased mental stress, which is predictive of depression (Hammen, 2015; Yi Wen & Mohd Aris, 2020). Meanwhile, the ATR is effective in diagnosing patients with dementia and cognitive decline (Baik et al., 2022; Jaramillo‐Jimenez et al., 2021; Özbek et al., 2021; Schmidt et al., 2013; Zawiślak‐Fornagiel et al., 2023); however, the results of this study suggest that depression may not be associated with this index. This difference might be attributed to a relatively lower degree of cognitive impairment in depression than in dementia. Moreover, a significant difference in ABR was observed across all electrode ranges, including the anterior frontal, posterior frontal, central, parietal, and occipital lobes. This global decrease in the ABR may be related to a wide range of alterations in functional connectivity in the resting state of depression (Helm et al., 2018; Mulders et al., 2015). Depression broadly affects myriad functional networks in the resting state, including the default mode, salience, and central executive networks, as well as the interaction between these networks and between the anterior and posterior default mode network (Mulders et al., 2015). Furthermore, more local analyses on functional connectivity have reported that not only are the anterior frontal, posterior frontal, parietal, occipital, and temporal lobes differently connected to each other, but also several subcortical and limbic regions that could affect the amplitudes of local electrodes are also associated with these alterations in functional connectivity (Helm et al., 2018). These alterations in functional connectivity might ultimately lead to global changes in the ABR. Beyond this global change in EEG power over every region, the central, frontal, and parietal lobes were highly altered, as evidenced by an AUC discrimination score >0.7 in the central, frontal, and parietal ABR, changes in the central and parietal TBR, and identification of the central ABR as the sole factor that significantly impacted BDI and STAI scores and depression probability. These significant localized differences may be attributed to the prominently altered functional connectivity around the medial frontal lobe, sensorimotor cortex, superior parietal lobe, and inferior parietal lobe (Helm et al., 2018; Mulders et al., 2015).

Among the three EEG power ratios, the ABR was the most effective biomarker of depression. Although the central and parietal TBRs exhibited differences between the depression and control groups, global TBR was not effective in diagnosing depression using machine learning. Meanwhile, each lobar ABR exhibited differences between the two groups, and the global ABR was more effective than TBR in diagnosing depression. However, the effectiveness of the ABR in diagnosing depression requires further investigation as the depression group in the current study was based on a dimensional approach and BDI score, which is not a reliable gold standard. . In addition, the EEG data used in the current study were extracted from young populations underage of 25, but depressive disorders are most frequent between 25 and 65 (Gurland, 1976). A paucity of sample sizes and variability in FFT parameters might have led to an over‐ or underestimation of the results of the current study. Hence, further analyses with bigger sample sizes and various cognitive tests with diverse age groups are needed to evaluate the real effectiveness of the ABR in diagnosing depression. Moreover, current diagnostic accuracy in this study may not be enough for practical and clinical use, although high discrimination scores still imply that ABR and TBR can be potential markers of depression. Deep learning method with an appropriate classifier would further improve the diagnostic accuracy using spectral power ratios. We believe that the results of this study provide insights for future work on a more effective and efficient diagnostic model of depression.

## AUTHOR CONTRIBUTIONS

Jinwon Chang designed and led the project, analyzed the data, and wrote the manuscript. Yuha Choi prepared and reviewed the manuscript. All authors revised and approved the content of the manuscript, contributed to the article, and approved the submitted manuscript.

## CONFLICT OF INTEREST STATEMENT

The authors declare no conflicts of interest.

### CONSENT STATEMENT

All participants provided written informed consent.

### PEER REVIEW

The peer review history for this article is available at https://publons.com/publon/10.1002/brb3.3173.

## Data Availability

The data that support the findings of this study are openly available in the OpenNeuro database (doi:10.18112/openneuro.ds003478.v1.1.0).
